# Differentiation of patients with mild cognitive impairment and healthy controls based on computer assisted hand movement analysis: a proof-of-concept study

**DOI:** 10.1038/s41598-022-21445-4

**Published:** 2022-11-09

**Authors:** Andras Attila Horvath, Dalida Borbala Berente, Balazs Vertes, David Farkas, Gabor Csukly, Tom Werber, Janos Andras Zsuffa, Mate Kiss, Anita Kamondi

**Affiliations:** 1 Department of Anatomy Histology and Embryology, Semmelweis University, Budapest, Hungary; 2 Neurocognitive Research Center, National Institute of Mental Health, Neurology and Neurosurgery, 57 Amerikai út, 1145 Budapest, Hungary; 3 School of PhD Studies, Semmelweis University, Budapest, Hungary; 4 Precognize Ltd, Budapest, Hungary; 5 Moholy-Nagy University of Art and Design, Budapest, Hungary; 6 Department of Psychiatry and Psychotherapy, Semmelweis University, Budapest, Hungary; 7 Department of Family Medicine, Semmelweis University, Budapest, Hungary; 8 Siemens Healthcare, Budapest, Hungary; 9 Department of Neurology, Semmelweis University, Budapest, Hungary

**Keywords:** Health care, Neurology

## Abstract

Mild cognitive impairment (MCI) is the prodromal phase of dementia, and it is highly underdiagnosed in the community. We aimed to develop an automated, rapid (< 5 min), electronic screening tool for the recognition of MCI based on hand movement analysis. Sixty-eight individuals participated in our study, 46 healthy controls and 22 patients with clinically defined MCI. All participants underwent a detailed medical assessment including neuropsychology and brain MRI. Significant differences were found between controls and MCI groups in mouse movement characteristics. Patients showed higher level of entropy for both the left (F = 5.24; p = 0.001) and the right hand (F = 8.46; p < 0.001). Longer time was required in MCI to perform the fine motor task (p < 0.005). Furthermore, we also found significant correlations between mouse movement parameters and neuropsychological test scores. Correlation was the strongest between motor parameters and Clinical Dementia Rating scale (CDR) score (average r: − 0.36, all p’s < 0.001). Importantly, motor parameters were not influenced by age, gender, or anxiety effect (all p’s > 0.05). Our study draws attention to the utility of hand movement analysis, especially to the estimation of entropy in the early recognition of MCI. It also suggests that our system might provide a promising tool for the cognitive screening of large populations.

## Introduction

Cognitive impairment due to major neurocognitive disorders (NCDs) such as Alzheimer’s disease is a growing public health concern. According to estimations, NCDs are going to represent the first cause of mortality and morbidity among the elderly by 2050^1^. Evidence from neuropathological and neuroimaging studies suggests that structural and functional changes of the nervous system precede the appearance of the first cognitive symptoms by decades^2,3^. It has also been accepted, that during this prodromal phase various non-cognitive symptoms, like sleep disorders, depression, behavioral and personality changes, sensory and motor deficits might be identified^4^. Early identification of prodromal phases could provide possibility for timely counseling for patients and caregivers about early non-pharmacological interventions and potential treatment options^5^. Missed or delayed recognition of the early phase of cognitive decline may compromise the diagnosis of reversible forms of dementias and the diagnosis of treatable comorbidities. Dementia in the disease continuum is preceded by mild cognitive impairment (MCI). Individuals with MCI are still able to maintain independent social functioning and carry out everyday activities^6^. Interventional studies propose that lifestyle improvements together with proper drug therapies might reduce the risk of further cognitive impairment in patients with MCI^7^.

Despite the key importance of early identification of MCI, there are substantial obstacles to its timely diagnosis. This is mostly due to the limited time of practitioners for screening and because the value of MCI’s early detection is not completely clarified among the clinicians^8,9^. Moreover, many of the affected people believe that their early symptoms are due to aging, and they only visit their doctors once symptoms are getting worse. It has been reported that MCI and mild dementia is undiagnosed in half of the cases in the United States and in Europe^10,11^. A possible reason for this is the limited availability of screening procedures. While there are numerous paper-and-pencil tools suitable for screening (e.g., clock drawing test, Mini-cog, 6-item Cognitive Impairment Test)^12–14^ and for the diagnostic support of MCI (e.g., Montreal Cognitive Assessment, Saint Louise University Mental Status)^15,16^ they require trained staff for their administration and evaluation as well as the physical presence of the evaluated patient. Digital healthcare solutions might provide broader access to medical screening tools for larger populations^17^. Recently there has been a remarkable growth in the number of electronic cognitive tools with different acquisition settings for clinical or at-home use (e.g., E-MoCA, CogState)^18,19^. While these solutions provide ability for screening and repeated measurements, the use of these complex cognitive test batteries by elderly populations could be challenging due to lack of familiarity with digital technologies^20^. Intrinsic cultural bias, practice and ceiling effect and rater dependency could also compromise the validity of cognitive tests^21^. Another problem could be the relative nonadherence of the older adults within home care settings due to technical (e.g., different hardware background for various tests), logistical (e.g., exhaustive, time-consuming tests), physiological (e.g., vision problems) and cognitive (e.g., complicated test regime) issues^22,23^. Thus, a design of an automated, self-administered test battery consisting of simple tasks and targeting few cognitive subdomains only might represent an important direction for regular screening of large populations.

Visuo-motor (VM) abilities could be ideal candidates for these developments, since growing number of reports suggest impairment of VM performance already in early MCI^24,25^. VM skills are crucial human integrative cognitive processes that allow us to react to visual stimuli with a motor action. Writing, drawing, copying, walking is all VM tasks. Some of them involve large muscle groups, others are related to complex activation of small muscles generating fine movements. It has been proposed that fine VM ability-based computerized tasks could identify subtle cognitive impairment in visuomotor coordination that would otherwise not be detected^26^. Furthermore, detectable VM impairments (e.g., slowing in the required task time, decreased speed and velocity of motor actions) are present even in individuals at increased risk for AD before any objective clinical symptoms of dementia^26,27^. This finding suggests that VM ability-based tasks could be used to signalize early signs of cognitive decline before it becomes apparent on standardized neuropsychological tests^27^. Interestingly, the fine coordination and continuity of motor actions is less frequently analyzed in the previously described experiments. Shannon entropy might be an ideal tool to measure the high-order coordination of motor performance since the signal gathered from fine motor movements is the result of complex interactions of physiological processes, which entail complex fluctuations in the signal (i.e., random small muscle twitches’ effect on the signal while moving the mouse). These fluctuations give rise to uncertainty in the continuous signal recording, which can be measured by Shannon’s entropy.

Based on the above, we have developed an automated computer assisted hand movement analysis system to test VM abilities in the elderly population with a potential for self-administration. The aim of our present work was to determine the discriminative power of small amplitude hand movement characteristics in the differentiation of healthy and MCI population in a clinical setting with a proof-of-concept study.

## Methods

### Participants

Participants were recruited from the AlzEpi Cohort Observational Library (ACOL database) of the National Institute of Mental Health, Neurology and Neurosurgery, Budapest, Hungary. The cohort consists of healthy elderly individuals, and patients with MCI or dementia. Their diagnosis was established by a multidisciplinary team. The library is part of the Euro-Fingers international database (http://www.eufingers.com) and includes biometric, demographic, clinical, neuroimaging, neurophysiologic, neuropsychologic and CSF data. Every subject was native Hungarian.

In the current experiment, only right-handed, regular internet and email user participants were recruited. Individuals with dementia were not included in our research. Participants with the following risk factors of cognitive decline were not included: hypothyroidism, renal insufficiency, liver disease, vitamin B12 deficiency, alcohol or substance abuse, use of psychoactive drugs which influence cognitive function, demyelinating conditions, clinically important brain lesions (stroke, white matter lesions), head injury accompanied by loss of consciousness, major depression, schizophrenia, electroconvulsive therapy, hydrocephalus, syphilis, HIV infection or previous central nervous system infections. Participants with conditions possibly affecting the motor control of upper limbs (including tremor disorders, Parkinson’s disease or Parkinsonism, motoneuron disorder, lesions in cortical motor areas, neuromuscular disorders, cervical spinal cord disease, damage of peripheral nerves of upper limb) were not recruited either.

Participants were categorized into two groups. The subjects in the healthy control (HC) group had negative neurological status, and their neuropsychological examination excluded cognitive impairment. Structural brain magnetic resonance imaging (MRI) did not show significant lesions or cortical atrophy. The patient group comprised the MCI patients. Their diagnosis was based on the revised Petersen criteria^6^. The presence of cortical thinning of the entorhinal cortex, and the reduction of total grey matter volume were confirmed by MRI. MRI data were extracted according to a previously published procedure^28,29^. Objective decline in cognitive performance was reinforced by neuropsychological test battery as detailed below. All research activities took place at the National Institute of Mental Health, Neurology and Neurosurgery, Budapest, Hungary. Informed written consent was obtained from every participant. Each research activity was performed in accordance with the relevant guidelines and regulations. Our research was authorized by the Hungarian Medical Research Council (reference number: (024505/2015/OTIG).

### Neuropsychological evaluation

Each participant was evaluated by a neurologist, neuropsychologist, or trained neuroscientist. Our test battery included various neuropsychological tests. The Hungarian version of Rey Auditory Verbal Learning Test (RAVLT)^30^ was used to appraise subjective memory complaints. RAVLT is considered a highly sensitive test in the detection of MCI^31^. In the test participants are asked to attentively listen to and memorize 15 words (list A) read aloud by the examiner then repeat as many words as they can remember. The same process is repeated another four times (five recalls in total). The sum of the correctly recalled words during the five repetitions gives the immediate recall value (RAVLT Sum5) with a maximum possible score of 75. Then the participant is presented the same task, this time with a different list (list B) and only once. Immediately after that, the participant must recall list A. To determine the delayed recall score (RAVLT 7) thirty minutes later the participant is asked again to recall list A. The sum of correct words gives RAVLT 7 with a maximum of 15 items. In our research the Hungarian version of Addenbrooke’s Cognitive Examination (ACE)^32,33^ was chosen to assess global cognitive function. ACE includes the Mini Mental State Examination and subscores of various cognitive domains^34,35^. Trail Making Test A (TMT-A) was selected to assess attention and cognitive function while part B (TMT-B) was applied to estimate cognitive flexibility^36^. To complete TMT-A, participants are asked to connect circled numbers in an ascending order. In TMT-B, the task is to connect circled numbers in an ascending order and circled letter in alphabetic order alternately. Since increased level of anxiety and depression might worsen cognitive functions, we used the Hungarian version of Spielberger State and Trait Anxiety Inventory (STAI)^37^ and Beck Depression Inventory II (BDI-II)^38^. State version of STAI was applied to measure anxiety level before the experiment (state anxiety: STAI-S) and trait version of STAI to estimate general level of anxiety (trait anxiety: STAI- T). STAI-S was administered immediately before the visuo-motor paradigm. The 13-question long version of Beck Depression Inventory (BDI-13) was administered to describe the mood. BDI scores < 13 indicate minimal depression, 14–19 range represents mild, the range 20–28 moderate and a score higher than 29 signals severe depression. To assess social functioning and independence, Clinical Dementia Rating (CDR) Sum-of-Boxes score was administered by the examiner^39^. CDR analyzes six functions as memory, orientation, judgement, and problem solving, community affairs, home and hobbies and personal care with a 5-point scale where 0 means no impairment, 0.5 mild impairment, 1 moderate impairment, 2 severe impairment, 3 loss of independent functioning.

### Motor paradigm and data extraction

The Precognize system was developed by Precognize Ltd. in collaboration with medical specialists and researchers of the National Institute of Mental Health, Neurology and Neurosurgery within the National Brain Research Program II in the years of 2017–2021. The main feature of the program is the precise recording of mouse movements while the participants are completing simple tasks on the computer not focusing on their mouse “handling” but more on to finish the tasks at hand. The Precognize system applies research codes for the identification of patients and researchers to ensure that no personal data will leave the premises of the hosting institution. As soon as the program begins, these codes need to be filled by the research lead, but after this only participants’ mouse movements are allowed as from this moment on the computer mouse movements are recorded. On the first screen, the program greets the research participants and explains that they will have to solve three tasks. There is a trial version of each task, where the task must be solved in a simplified way, thus ensuring that the participant understands the task. The mouse was set so that the participants could activate the primary mouse button with index finger during both the right and the left-handed sample taking.

The development of the task was motivated by the structure of TMT. During the task, nine circled Arabic numerals (1–9) are shown on the screen in a fixed position. The task for the participant is to click on the numbers using the computer mouse, starting with 1 in ascending order as quickly as possible (1-2-3-4-5…). In the event of an error (outside click or wrong order), the program will not allow the participant to proceed, until a correct mouse click is performed. During data acquisition, the same mouse and laptop were used with the same setup. We also applied an ergonomic protocol ensuring that for data collection only the same chair, table and mousepad were used. Participants were tested at the same time each day, between 4 and 6 p.m. We took a left and a right-handed sample from each participant.

Mouse movement data were stored as log files. Movement parameters were extracted for both hands individually and separately for all subsections of the tasks. Subsections were defined as the period between the Arabic numbers (connecting the lower to the higher). Altogether 18 subsections were defined for all participants (9 for left hand and 9 for right hand). Finally, the computed parameters were averaged for the 9 subsections. Six movement parameters were calculated as distance, entropy, number of tries, time, speed, and velocity. The explanation and calculation of these parameters is detailed in Table 1.

**Table 1:** Description of mouse movement parameters.

Motor parameter	Description	Formula
Distance	The overall distance from the mouse movements	$\sum_{i=1}^{n}\sqrt{{\left({x}_{n}-{x}_{n-1}\right)}^{2}+{({y}_{n}-{y}_{n-1})}^{2}}$
Entropy	The Shannon entropy of the mouse movements, where the underlying distribution (random variable) is the two-dimensional coordinate of the location of the mouse on the screen	$-\sum_{i=1}^{n}{f(x;y)}_{i}\times lg{ f(x;y)}_{i}$
Number of tries	The sum of all mouse clicks	$\sum all \; clicks$
Time	The time required to complete the task	${t}_{n}-{t}_{0}$
Speed	The speed of the mouse movements	$\frac{distance}{time}$
Velocity	The velocity of the mouse movements	$\sum_{i=0}^{n}\frac{{distance}_{n}-{distance}_{n-1}}{{time}_{n}-{time}_{n-1}}$

*x* and *y* refer to *x* and *y* coordinates of the screen, while *t* refers to time.

### Data analysis

Distribution of data was analyzed with Kolmogorov–Smirnov test. Continuous variables of demographic and cognitive features were analyzed with independent samples t-test or Mann–Whitney U-test. Categoric variables were compared with Chi-squared tests. ANCOVA model was set to examine the influence of group effect (HC vs MCI as independent variable) on the fine-motor data (dependent variable), while removing the effect of age, gender, and state anxiety level (covariances). Since motor data might influence each other, we applied Benjamini–Hochberg correction for multiple comparisons. Effect sizes were estimated in Cohen’s d (0.2 < small effect, 0.5 < medium effect, 0.8 < large effect). Pearson-correlation was applied to analyze the association between neuropsychological performance and visuo-motor data showing significant differences in the intergroup comparisons represented by nominal p’s, suggesting their value as potential discriminative candidates.

## Results

### Demographic, clinical, and cognitive characteristics

Eighty individuals (31 MCI and 49 controls) of the databank matched the selection criteria. Twelve (9 MCI patients and 3 healthy individuals) declined the participation. Finally, sixty-eight individuals participated in our research, forty-six of whom belonged to the healthy control (HC) group while twenty-two belonged to the mild cognitive impairment (MCI) group. The HC group contained 27 females (58.70%) while the MCI group included 12 females (54.55%). Significant differences across the groups in the distribution of sex (p = 0.225) or in the length of education (p = 0.128) were not found. The two groups significantly differed in their age (F = 0.638; p = 0.019) and in some neuropsychological features including total ACE score (F = 60.086; p < 0.001), ACE letter fluency (F = 5.07; p = 0.028), ACE category fluency (F = 31.48; p < 0.001), RAVLT sum-5 score (F = 110; p = 0.015), RAVLT 7 score (F = 69.84; p = 0.008) and CDR score (Z = − 4.284; p < 0.001). MCI group also showed lower entorhinal thickness (F = 3.86, p = 0.017) and reduced grey matter volume (F = 4.21, p = 0.012) compared to HC. In other parameters, significant differences were not demonstrated (all p’s > 0.05). Demographic data and cognitive characteristic features are presented in Table 2.

**Table 2:** Demographic, clinical, and neuropsychological characteristics of the HC and MCI groups.

	HC (n = 46)	MCI (n = 22)	p-value	Effect size (Cohen’s d)
**Demographics**				
Age (years)^a^	66.76 ± 7.63	71.18 ± 5.82	0.019*	0.65
Sex (% of females)^b^	58.70	54.55	0.225	–
Education (years)^a,d^	15.54 ± 1.93	14.63 ± 2.38	0.128	0.42
**Neuroimaging**				
Average entorhinal thickness (mm^2^)^a,d^	3.43 ± 0.25	3.11 ± 0.23	0.017*	1.31
Total grey matter volume (mm^3^)^a,d^	576,828 ± 57,471	573,914 ± 57,471	0.012*	0.05
**Neuropsychology**				
MMSE^a,d^	28.76 ± 1.08	26.5 ± 1.79	0.088	1.55
ACE total^a,d^	93.93 ± 3.85	80.22 ± 9.7	< 0.001*	1.88
ACE orientation^a,d^	9.77 ± 0.53	9.19 ± 0.16	0.434	0.59
ACE attention^c,e^	8.00 (8.00–8.00)	8.00 (8.00–8.00)	0.307	0.45
ACE memory^a,d^	29.93 ± 0.25	23.7 ± 6.07	0.089	1.17
ACE letter fluency^a,d^	5.98 ± 1.39	4.29 ± 2.07	0.028*	0.97
ACE category fluency^a,d^	6.67 ± 0.04	4.91 ± 1.76	< 0.001*	1.36
ACE language^a,d^	27.93 ± 0.26	26.7 ± 1.88	0.388	0.96
ACE visuospatial^a,d^	4.8 ± 0.1	4.1 ± 0.73	0.135	1.2
RAVLT sum-5^a,d^	49.24 ± 6.36	29.2 ± 7.51	0.015*	2.93
RAVLT 7^a,d^	10.17 ± 2.73	3.82 ± 2.21	0.008*	2.59
TMT-A (in s)^a,d,f^	39.98 ± 11.76	85.6 ± 55.1	0.288	1.19
TMT-B (in s)^a,d,f^	87.83 ± 46.17	186 ± 117.7	0.078	1.12
CDR^c,e,f^	0 (0–0.2)	0.88 (0.4–1)	< 0.001*	1.19
BDI-13^a,d,f^	4.3 ± 4.02	5.61 ± 3.15	0.32	0.28
STAI-S^a,d,f^	36.5 ± 9.23	43.25 ± 8.38	0.32	0.77
STAI-T^a,d,f^	41.95 ± 9.51	42.38 ± 7.85	0.118	0.04

HC healthy control, MCI mild cognitive impairment, MMSE Mini-Mental State Examination, ACE Addenbrooke Cognitive Examination, RAVLT Rey Auditory Verbal Learning Test, TMT Trail-Making Test, CDR Clinical Dementia Rating scale, BDI Beck Depression Inventory, STAI-S Spielberger State and Trait Anxiety Inventory State Score, STAI-S Spielberger State and Trait Anxiety Inventory Trait Score.

*Indicates significant differences (p < 0.05).

^a^Data was analyzed with independent sample t-test.

^b^Data was analyzed with Chi-squared test.

^c^Data was analyzed with Mann–Whitney U-test.

^d^Data is given in the form of mean ± standard deviation (SD).

^e^Data is given in the form of median and interquartile ranges (IQ1–IQ3).

^f^Lower score associates with better cognitive performance.

### Inter-group comparisons of mouse-movements

**Figure 1:**
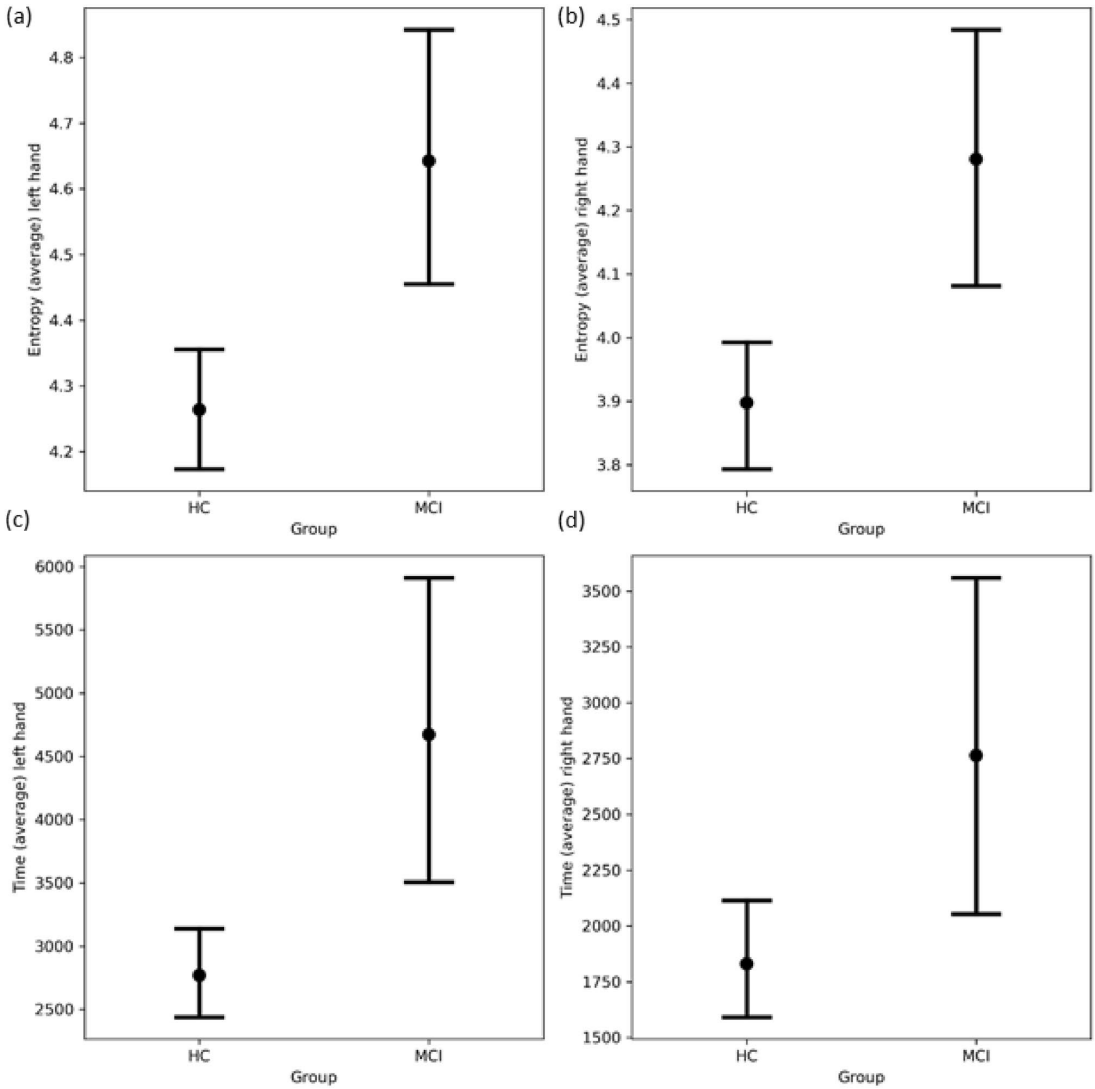
Error bars of intergroup comparisons of patients with MCI and healthy controls. Patients had significantly higher entropy in the movements and required longer time during the motor task. Difference is more characteristic in the left hand. All graphs demonstrate significant differences (p < 0.05). HC: healthy control; MCI: mild cognitive impairment.

We found significant differences between HC and MCI groups in the distance of left-hand movements (F = 1.16, p = 0.0134), in the time of movements with left hand (F = 4.32; p = 0.005) and in the entropy of the left-hand movements (F = 5.24; p = 0.001) (Fig. 1). The largest difference in the left-hand movements was presented in the entropy (largest F value and large effect size). Similar alterations were presented in the right-hand (dominant hand) movements including the distance (F = 1.03, p = 0.019) required time of movements (F = 4.626; p = 0.003) and entropy (F = 8.46; p < 0.001). The most prominent difference was detected in the entropy of right-hand movements (largest F value and large effect size) (Fig. 1). The differences survived the Benjamini–Hochberg correction for time and entropy (corrected p < 0.05), while significance disappeared for the distance (corrected p > 0.05). A non-significant trend was also visible in other motor parameters including more tries, decreased speed, and increased velocity in MCI patients. Exact characteristics are presented in Table 3. Sex, age, and state anxiety level did not have significant modifier effect (all nominal *p*’s > 0.05). Based on these estimations, distance, entropy, and duration seemed to be the best candidates for further correlation analysis. Interestingly, data of the left hand showed better potential in these parameters (Fig. 1), represented by the fact that effect sizes were larger than that of the dominant hand (Table 3).

**Table 3:** Differences in the motor movement characteristics among MCI and HC participants.

	HC (n = 46)	MCI (n = 22)	Nominal p-value	Effect size (Cohen’s d)
**Left hand**				
Average distance of 9 routes (pixel)	1611.94 ± 1044	2319.31 ± 1368.18	0.0134	0.58
Average entropy of 9 routes	4.26 ± 0.32	4.64 ± 0.47	0.001*	0.94
Average number of tries of 9 routes	2.88 ± 0.31	3.17 ± 0.69	0.329	0.54
Average speed of 9 routes (pixel/ms)	0.53 ± 0.14	0.45 ± 0.2	0.124	0.46
Average time of 9 routes (ms)	2932.41 ± 1601.92	4672.21 ± 2648.63	< 0.005*	0.79
Average velocity of 9 routes (pixel/ms)	4,911,765 ± 9,621,912	7,765,693 ± 7,971,712	0.389	0.32
**Right hand**				
Average distance of 9 routes (pixel)	1181.41 ± 275.2	1714.32 ± 591.35	0.0119	0.22
Average entropy of 9 routes	3.9 ± 0.35	4.28 ± 0.48	< 0.001*	0.9
Average number of tries of 9 routes	3.2 ± 0.62	3.66 ± 1.76	0.97	0.34
Average speed of 9 routes (pixel/ms)	0.57 ± 0.17	0.47 ± 0.19	0.069	0.55
Average time of 9 routes (ms)	1830.25 ± 901.93	3545.95 ± 3064	0.003*	0.75
Average velocity of 9 routes (pixel/ms)	2,462,435 ± 8,318,595	2,923,976 ± 5,945,440	0.5	0.06

The required time, the distance route and the entropy were significantly increased in the MCI group. p values are represented as nominal p’s, while * indicates significant differences following Benjamini–Hochberg correction for multiple comparisons.

HC healthy control, MCI mild cognitive impairment, ms millisecond.

### Correlation analysis between mouse-movement parameters and neuropsychological performance

**Figure 2:**
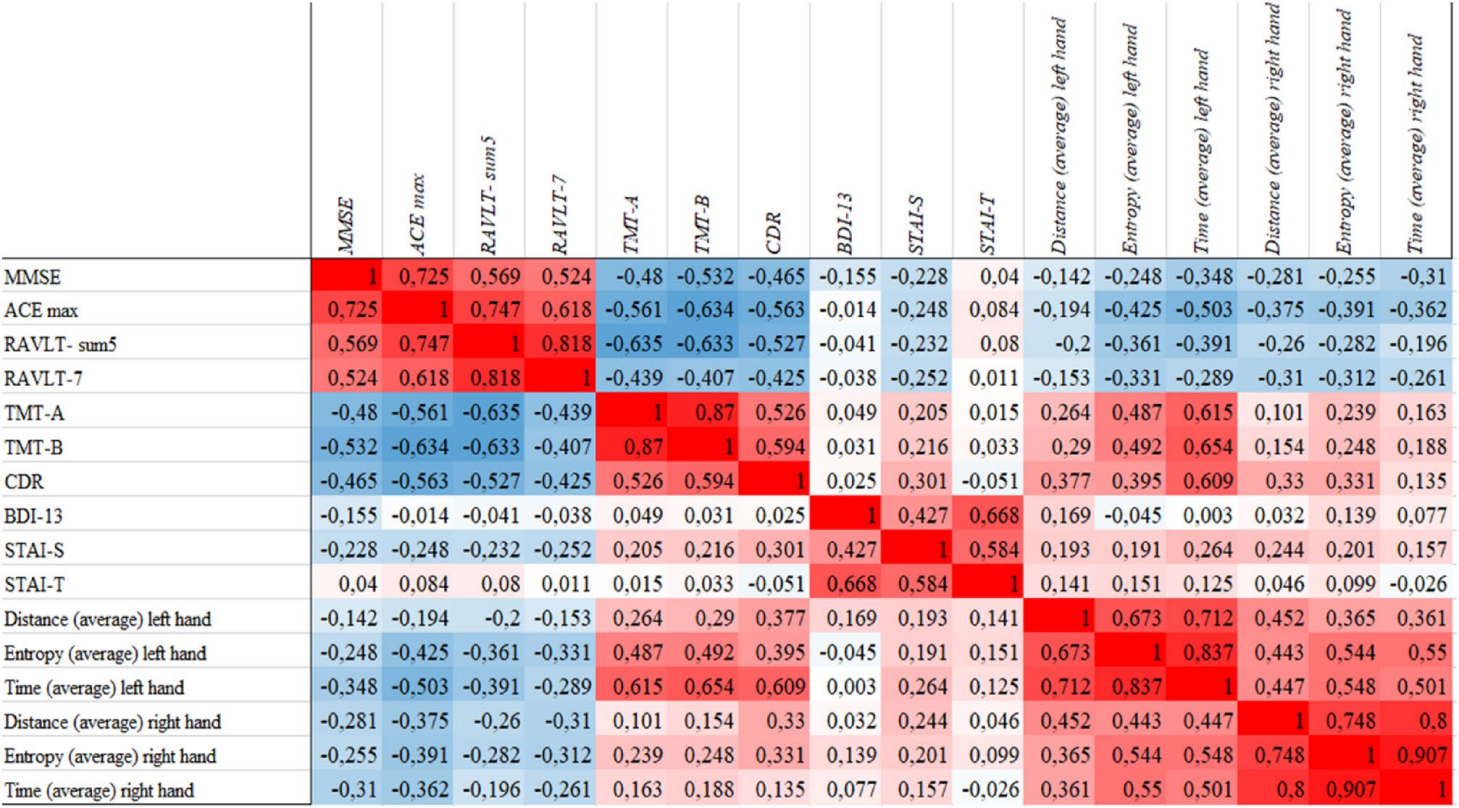
Corrgram between hand movement parameters and neuropsychological test results. Blue color indicates negative correlations, while red demonstrates positive correlations. A clear trend is visible, patients with lower cognitive scores in MMSE, ACE, RAVLT have higher movement values (negative r). However, lower cognitive scores are also indicated with higher TMT and CDR scores, where mouse movements show also higher values (positive r). In conclusion, increase in motor parameters indicates worse cognitive performance. Highest r values are presented in time of left-hand movements. A trend is also visible for higher r values of other movement parameters in case of the left hand. MMSE Mini-Mental State Examination, ACE Addenbrooke Cognitive Examination, RAVLT Rey Auditory Verbal Learning Test, TMT Trail-Making Test, CDR Clinical Dementia Rating scale, BDI Beck Depression Inventory, STAI-S Spielberger State and Trait Anxiety Inventory State Score, STAI-T Spielberger State and Trait Anxiety Inventory Trait Score.

Results of Pearson-correlation analysis between the extracted mouse movement parameters and neuropsychological performance are presented in Table 4 (p-values) and Fig. 2 (r coefficients). Mouse movement parameters showed strong and significant association with the neuropsychological test results including MMSE, ACE, RAVLT, TMT and CDR.

**Table 4:** Significance results (p-values) of correlation analysis across hand movement parameters and neuropsychological performance.

	Left hand: distance (pixel)	Left hand: entropy	Left hand: time (in ms)	Right hand: distance (pixel)	Right hand: entropy	Right hand: time (in ms)
MMSE	0.064	0.006*	0.006*	0.002*	0.003*	0.013*
ACE total	0.026*	< 0.001*	< 0.001*	< 0.001*	< 0.001*	0.003*
RAVLT SUM-5	0.037*	0.001*	0.002*	0.007*	0.004*	0.121
RAVLT 7	0.097	0.002*	0.025*	0.002*	0.002*	0.037*
TMT-A (in s)^a^	0.009*	< 0.001*	< 0.001*	0.129	0.009*	0.203
TMT-B (in s)^a^	0.022*	< 0.001*	< 0.001*	0.225	0.046*	0.139
CDR^a^	< 0.001*	< 0.001*	< 0.001*	< 0.001*	< 0.001*	< 0.001*
BDI-13^a^	0.074	0.784	0.984	0.319	0.081	0.544
STAI-S^a^	0.321	0.373	0.047	0.217	0.370	0.227
STAI-T^a^	0.280	0.241	0.356	0.719	0.437	0.842

Numerous significant correlations were presented between test scores measuring cognitive performance and motor actions, while there was no significant correlation among movements and neuropsychiatric properties including mood and anxiety measurements. *Indicates significant correlation (p < 0.05).

MMSE: Mini-Mental State Examination; ACE: Addenbrooke Cognitive Examination; RAVLT: Rey Auditory Verbal Learning Test; TMT: Trail-Making Test; CDR: Clinical Dementia Rating scale; BDI: Beck Depression Inventory; STAI-S: Spielberger State and Trait Anxiety Inventory State Score; STAI-S: Spielberger State and Trait Anxiety Inventory Trait Score.

^a^Lower score associates with better cognitive performance.

However, correlation was not significant between movement parameters, anxiety, and mood measures such as STAI-S, STAI-T, BDI-13.

**Figure 3:**
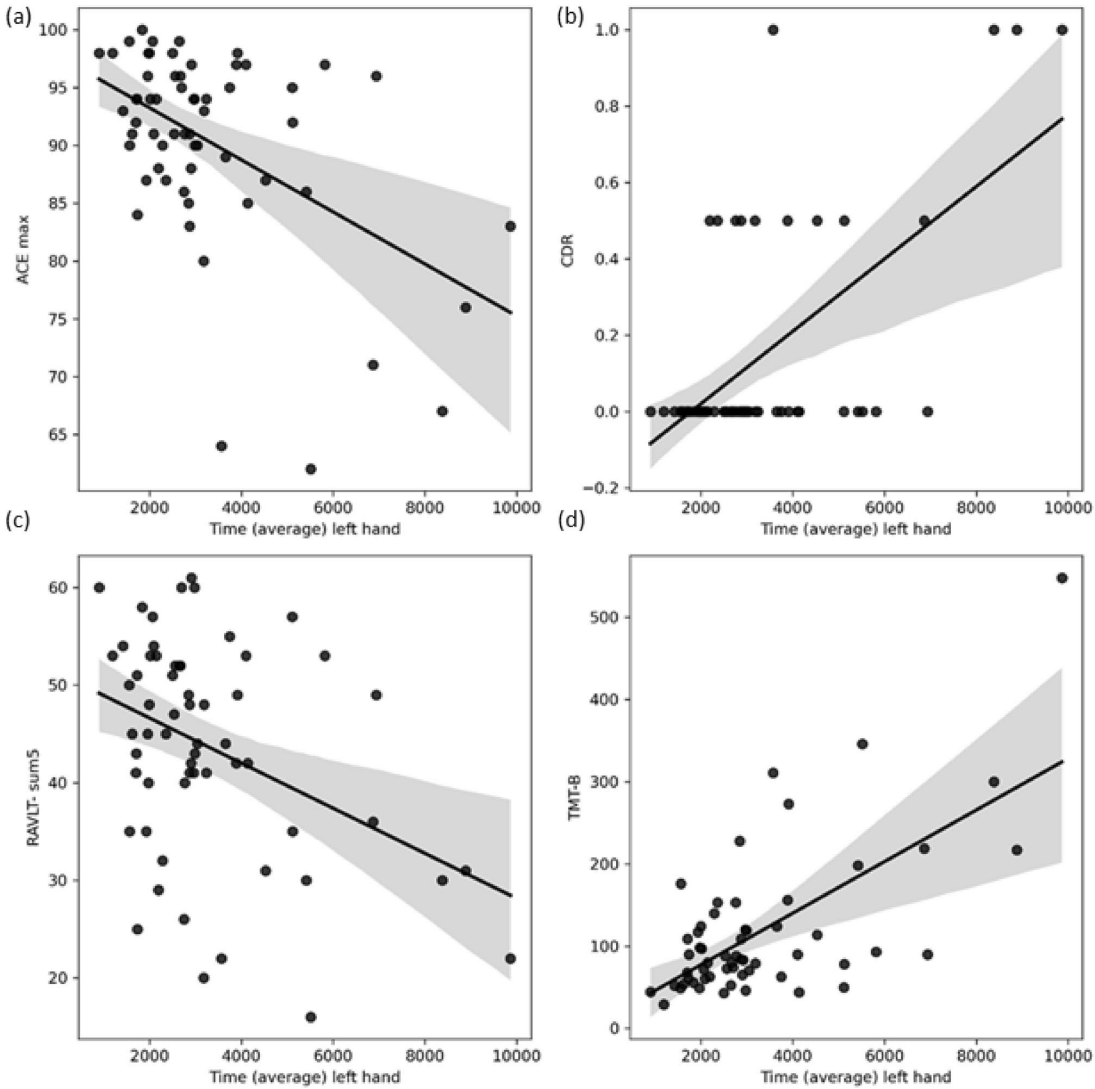
Scatter dots of correlation analysis across time average of left-hand movements (in ms) and neuropsychological test results. Where worse cognitive performance is characterized by lower cognitive scores (Addenbrooke Cognitive Examination (ACE) ranging between 0 and 100 and Rey Auditory Verbal Learning (RAVLT) summary of first 5 trials ranging between 0 and 75), negative correlation is presented with the time required for mouse movements. However, where worse cognitive performance associates with higher neuropsychological scores (Clinical Dementia Rating Scale (CDR) ranging between 0 and 3 and Trail-Making Test B (TMT-B) measured in required seconds), positive correlation is found. ACE Addenbrooke Cognitive Examination, RAVLT Sum-5 Rey Auditory Verbal Learning Test Summary of first 5 trials. TMT-B Trail-Making Test B, CDR Clinical Dementia Rating scale.

By measures where lower scores indicate worse cognitive status (MMSE, ACE, RAVLT Sum-5, RAVLT 7), negative correlation with the motor parameters were demonstrated in all cases (*r* values ranged between − 0.14 and − 0.5). The strongest association was found between movement features and ACE total score (average r: − 0.37, all p’s < 0.05). Time with left hand movements seemed to be the best predictor of cognitive measures in this test series (average r: − 0.38, all p’s < 0.05) (Fig. 3), while entropy was the second best predictor considering r values (average − 0.34 for left hand and − 0.31 for right). The two hands did not differ significantly in the r values (average r for left hand is − 0.29 vs average r for right hand is − 0.29).

In the analysis of neuropsychological test results where lower scores indicate better cognitive performance (TMT-A, TMT-B, CDR), positive correlation was detected in all cases with the movement parameters (r values ranged between + 0.1 and + 0.65). The strongest association was present between movement features and the CDR score (average r: − 0.36, all p’s < 0.001). Time with left hand movements seemed to be the best predictor of cognitive skills in this test series (average r: + 0.62, all p’s < 0.001) (Fig. 3), while entropy was also prominent (average r for left hand: + 0.49). A clear trend was also visible that left hand movements had significantly larger r values (average r for left hand is 0.46 vs average r for right hand is 0.21) (Fig. 2).

## Discussion and conclusion

In this study, we present the first results obtained with a custom-made digital diagnostic system; the Precognize. In our experiment, we determined the utility of this automated, fine-motor test in the recognition of MCI. Our patient group consisted of a multidomain MCI population based on their traditional neuropsychological test results (ACE, CDR, RAVLT, TMT) (Table 1). While memory impairment was the most characteristic hallmark of our patients (largest effect size is present in the scores of RAVLT), significantly reduced cognitive functions were also depicted in verbal fluency, global cognitive scores, executive functions (Table 2). According to the literature, MCI patients have lower ACE scores^40^, RAVLT scores^28,41^ and elevated CDR scores^42^, as we also demonstrated in our sample. As a reinforcement of primary neural loss, MCI patients had significantly decreased grey matter volume and entorhinal thickness, as a typical neuroimaging hallmark of MCI^43^. Furthermore, the extensive exclusion criteria of potential causes of cognitive impairment are potential selection bias supporting that our patients might had MCI exclusively due to neurodegenerative disorders instead of reversible factors. It might indicate that our approach is a potential diagnostic tool for the early recognition of neurodegenerative disorders instead of the exhaustive screening of the entire MCI population. Further follow-up studies are needed to support the impact of these selection criteria on the utility of the method in different MCI patient populations.

During the mouse movement task, right-handed, regular internet user participants were asked to connect 9 Arabic numbers in ascending order using an electronic mouse Participants performed the test with their right and their left-hand as well. The entire procedure took less than 5 min. We extracted 6 movement features (Table 1) (distance, duration, velocity, speed, entropy, and the number of clicks or tries) and compared the data of healthy individuals to patients with MCI. In the intergroup comparisons, we revealed that MCI patients required significantly longer time to perform the protocol and showed higher level of entropy during the movements (Table 3, Fig. 1). Interestingly, the left-hand data showed better discriminative values represented by larger effect sizes. In general, effect sizes for movement differences were in the large range (0.8 <), suggesting robust discriminative potential. We also analysed the potential modifier effect of age, sex, and anxiety level on the motor performance but none of these factors had significant effect. We also extracted the background of intergroup differences using correlation measures to estimate the association of discriminative movement features and neuropsychological test results (Table 4, Fig. 2). Correlation analysis revealed that participants who had better global cognitive functioning proved by higher ACE and MMSE scores or better memory performance demonstrated in RAVLT scores needed less time to perform the protocol and showed reduced entropy during the actions (negative r values) (Fig. 3). The association was mild or moderate based on the r values, however it was constantly moderate with the ACE total score. Since ACE is frequently applied in the diagnosis of cognitive impairment, our results seem to represent the potential value of our digital diagnostic system. As a reinforcement, we also showed that subjects having worse daily functioning (higher CDR score) and reduced cognitive speed and flexibility (higher TMT scores) showed longer time and higher level of entropy (positive r values) (Fig. 3). The correlation was moderate. Superiority of left hand was also demonstrated in the correlation analysis (higher r values). Noticeably, significant correlation was not present between test values representing anxiety (STAI) and mood (BDI) level and motor performance (Table 4).

Slowing in motor performance and psychomotor abilities is in line with the current MCI literature^44^. In our experiment, slowing is characterized as an increase in the required time for the task. While it is a known phenomenon, its use as an automated diagnostic marker might have important further applications. Interestingly, while Shannon entropy of movements is a barely analysed feature in neurocognitive studies^45^, it seemed to be the best predictor of cognitive impairment in our sample (largest effect size in intergroup comparisons). A higher value of entropy in MCI population could signal that the test subject’s mouse movement characteristics was “unexpected”, meaning, it deviated greatly from the expected route^46^. Some studies showed that analysis of entropy could reveal characteristic features of polyrhythmic, rapid aimed movements in healthy individuals^47,48^. Others applied it to describe personal eye movement variations^49^. These reports suggest that analysis of movement entropy can characterize individual motor characteristics and higher level of entropy associate with a more disharmonized motor control. Based on these observations, it is intriguing to measure its utility in motor disorders. Some studies showed that entropy analysis of drawing (Archimedes’ spiral, copying of pentagons) can sensitively discriminate essential tremor and Parkinson patients from healthy controls^50,51^. In Alzheimer spectrum disease, movement entropy seems to be a barely applied analysis method. We found only one study analysing movement entropy in MCI showing that increased entropy obtained from gait kinematics depicts cognitive decline in the early phase with high sensitivity^52^. To our knowledge, our study is the first demonstrating the potential utility of hand movement entropy as a diagnostic marker of MCI.

While numerous electronic cognitive tasks are being tested for the detection of MCI^22^, analysis of small amplitude motor actions are less common. The major aim behind the application of movement analysis as cognitive screening is that traditional electronic cognitive tests require long administration time (~ 30 min in average) and the results are strongly influenced by pre-evaluation conditions such as emotional, mental tiredness or increased anxiety of the participant^22^. The major complication with fine motor tests is the large diversity which makes generalization difficult. Some authors applied visuo-motor paradigms using sensitive motion sensors and eye trackers^53,54^ and showed high discriminative potential in the recognition of MCI. Other studies applied fine motor actions using a touch screen and showed high classification accuracy between Alzheimer patients and HC. While these studies are similar to our approach regarding methodologies, they did not test the discriminative potential and classification accuracy in MCI patients^24,27^. A similar study approach examined 19 pre-AD patients vs 47 HCs and found higher response time in patients with the application of a computer based visuo-motor paradigm^26^. Some studies have been published also on the kinematic analysis of handwriting^55,56^. In hand-writing experiments, it is an established common finding that aging^57^ and anxiety have a strong modifier effect on the movement parameters^58^. As a summary, we can state that the limitation in most of these studies as screening methods is the complicated setup of the diagnostic system, sometimes the strong financial need, and the modifier effect of anxiety or age. In contrast, these factors represent the strength of our approach: (1) the hardware background is widely available and cheap; (2) the examination period is less than 5 min; (3) anxiety, mood, age, or gender did not impact the motor parameters; (4) the lack of cultural and language bias.

The limitation of our study is similar to other movement analysis protocols used in the diagnosis of MCI: (1) the low sample size; and (2) the lack of validation on independent databases. To overcome these difficulties, we plan to expand the analysis to a population wide experiment using various databanks.

In conclusion, our proof-of-concept phase experiment shows promising potential of the Precognize system in the early recognition of MCI. The strength of this approach is the low time needed to perform the test and the cost-effectiveness of this system. Our results also reinforce previous data showing the early impairment of fine-motor control in MCI. We also draw attention to the potential application of movement entropy characterisation in the early recognition of cognitive decline. Analysis of fine movements might provide an ideal battery for cognitive screening of large populations due to the potential for automatization, self-administration, and potential application of artificial intelligence solutions for large population studies as well.

## Data Availability

Raw data are available upon request to the corresponding author.
